# Cost of clinical events in health economic evaluations in Germany: a systematic review

**DOI:** 10.1186/1478-7547-10-7

**Published:** 2012-05-31

**Authors:** Monika Scheuringer, Narine Sahakyan, Karl J Krobot, Volker Ulrich

**Affiliations:** 1 Outcomes Research Department, MSD Sharp & Dohme GmbH, Haar, Germany; 2 Department of Medical Informatics, Biometry, and Epidemiology, Ludwig Maximilians University Munich, Munich, Germany; 3 Division of Public Health Decision Modelling, Health Technology Assessment and Health Economics, ONCOTYROL - Center for Personalized Cancer Medicine, Innsbruck, Austria; 4 Institute of Public Health, Medical Decision Making and Health Technology Assessment, Department of Public Health and Health Technology Assessment, UMIT- University for Health Sciences, Medical Informatics and Technology, Hall i.T., Austria; 5 Department of Law and Economics, Institute of Public Finance, University of Bayreuth, Bayreuth, Germany

**Keywords:** Costs, Diabetes mellitus, Germany, Health economics

## Abstract

Guidance from the Institute for Quality and Efficiency in Health Care (IQWiG) on cost estimation in cost–benefit assessments in Germany acknowledges the need for standardization of costing methodology. The objective of this review was to assess current methods for deriving clinical event costs in German economic evaluations. A systematic literature search of 24 databases (including MEDLINE, BIOSIS, the Cochrane Library and Embase) identified articles, published between January 2005 and October 2009, which reported cost-effectiveness or cost-utility analyses. Studies assessed German patients and evaluated at least one of 11 predefined clinical events relevant to patients with diabetes mellitus. A total of 21 articles, describing 199 clinical cost events, met the inclusion criteria. Year of costing and time horizon were available for 194 (97%) and 163 (82%) cost events, respectively. Cost components were rarely specified (32 [16%]). Costs were generally based on a single literature source (140 [70%]); where multiple sources were cited (32 [16%]), data synthesis methodology was not reported. Cost ranges for common events, assessed using a Markov model with a cycle length of 12 months, were: acute myocardial infarction (nine studies), first year, 4,618–17,556 €; follow-up years, 1,006–3,647 €; and stroke (10 studies), first year; 10,149–24,936 €; follow-up years, 676–7,337 €. These results demonstrate that costs for individual clinical events vary substantially in German health economic evaluations, and that there is a lack of transparency and consistency in the methods used to derive them. The validity and comparability of economic evaluations would be improved by guidance on standardizing costing methodology for individual clinical events.

## Background

In Germany, the Institute for Quality and Efficiency in Health Care (IQWiG) has been legally empowered to assess the increase in benefit as well as the relationship of benefits and costs for drugs and healthcare procedures. IQWiG has recently published a working paper on cost estimation in cost–benefit assessments in Germany [1]. In this document, it is acknowledged that there is potential for standardization of costing methodology to improve the comparability of health economic evaluations. Lists of agreed unit costs (so-called standard cost lists) that supplement guidelines for health economic break at evaluation in other countries (e.g. Australia [2], Canada [3,4], and the Netherlands [5]) are cited as examples of such standardization. In addition, the ‘Methods in Health Economic Evaluation’ Working Group (AG Methoden der gesundheitsökonomischen Evaluation [AG MEG]) of the German Society for Social Medicine and Prevention have developed an approach to standardizing cost estimates [6]. In general, however, such lists do not comprise agreed costs for frequently observed clinical events as estimates for the actual costs accrued (so-called costs for clinical events).

Costs for clinical events are essential for creating health economic models that are able to accurately simulate both the health outcomes and cost of different treatment options by incorporating evidence from a variety of sources. Ideally, the way in which these costs are obtained and the cost components included, should be described in the reporting of each study. We conducted a systematic literature review of costs for clinical events used in health economic evaluations in Germany from 2005 to 2009 in order to provide insight into the derivation of these costs and to assess the potential for providing future standard lists for clinical events.

## Methods

### Identification of relevant studies

A systematic literature review was conducted to determine the costs for clinical events that are relevant to patients with diabetes mellitus, according to Preferred Reporting Items for Systematic reviews and Meta-Analyses (PRISMA) literature review methodology [7]. Diabetes mellitus has a significant economic burden and it was assumed that an adequate number of health economic analyses in this disease area would have been published in the time frame of interest to enable an analysis of costs to be performed. Typically such health economic models include a range of disease stages and provide invaluable insight into the derivation of costs of clinical events. In total, 24 databases, including MEDLINE, BIOSIS, the Cochrane Library and Embase (see Additional file 1), were searched for articles published between January 2005 and October 2009. The reference lists of systematic reviews and meta-analyses identified were screened independently by two researchers to assess whether they included health economic evaluations that met the criteria for inclusion; disagreements were resolved after discussion between the two researchers. The final search was conducted on 30 October 2009.

**Figure 1:**
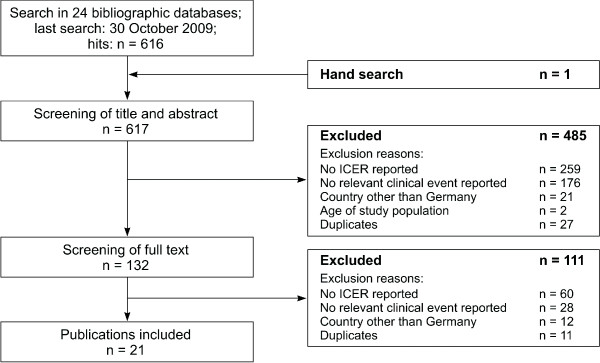
Flow chart of included studies.

Articles were included in this review if they: were economic analyses reporting an incremental cost-effectiveness ratio (ICER); included German patients aged at least 18 years; were published in 2005 or later in English or German; and evaluated at least one of 11 predefined clinical events (acute myocardial infarction [MI], stroke, angina pectoris, heart failure, microalbuminuria and/or macroalbuminuria, renal failure, cataract, retinopathy, blindness, neuropathy, and amputation owing to diabetic foot syndrome). Only full text published articles were included in the review; abstracts referencing congress presentations were excluded. The flow of studies through the systematic review is shown in Figure 1.

### Information extraction and analysis

In order to examine the ability of the extracted costs to inform future standard lists for clinical events, the characteristics of the health economic evaluations (type of economic evaluation, model type, time horizon, cycle length, target population, severity of health status, type of intervention, outcome measure, perspective of analysis, primary time horizon, secondary time horizon, type of sensitivity analysis), costs for clinical events, and derivation of these costs (time horizon covered, cost compounds covered, year of cost data, currency conversion rate, inflation rate) were extracted using a Microsoft Access database structured form. The maximum and minimum costs used in Markov models that had a cycle length of 12 months were then summarized in a table, with stratification by first cycle and follow-up cycles.

## Results

### Identification of relevant studies

In total, 616 articles were identified from database searches, and one further article was identified through hand-searching of reference lists from systematic reviews and meta-analyses. Of the 617 articles identified, 485 were excluded based on the title or abstract, and a further 111 articles were excluded after evaluation of the full text; 21 articles were therefore included in the final review (Figure 1).

### Study characteristics

The study characteristics of the included articles are described in Table 1. Ten articles reported cost-utility analyses, nine reported cost-effectiveness analyses, and two reported findings from both types of economic analysis. Overall, 19 analyses were undertaken from the payer perspective, one from the societal perspective, and in one article the perspective was not specified. Most articles reported the results of Markov (*n* = 12) or semi-Markov models (*n* = 5). Four articles reported results from a decision tree, two of which also included Markov modelling.

**Table 1:** Included studies

**Study**	**Patients**	**Intervention**	**Health states evaluated**	**Perspective**	**Time horizon**
Annemans et al. 2006 [8]	At risk of cardiovascular disease (primary prevention)	ASA	· MI	Payer	10 years
			· Stroke		
Berg et al. 2007 [9]	STEMI	Clopidogrel + ASA	· MI	Societal	1 year
			· Stroke		
Berg et al. 2008 [10]	ACS undergoing PCI	Clopidogrel + ASA	· MI	Payer	Not specified
			· Stroke		
Berger et al. 2008 [11]	Elevated risk of MI, ischaemic stroke	Clopidogrel	· MI	Payer	2 years
			· Stroke		
Brüggenjürgen et al. 2007 [12]	ACS without ST-elevation	Clopidogrel + ASA	· MI	Payer	Lifetime
			· Stroke		
Claes et al. 2008 [13]	Stroke	Dipyridamole + ASA	· Stroke	Payer	Lifetime
Gandjour et al. 2007 [14]	Hypertension at high or low risk for CVD	National hypertension treatment programme	· MI	Payer	Lifetime
			· Stroke		
Jürgensen et al. 2009 [15]	Dialysis	Immunosuppressive therapy – sirolimus	· End-stage renal disease	Payer	2 years
Lamotte et al. 2006 [16]	CVD	ASA	· MI	Payer	10 years
			· Stroke		
Lamotte et al. 2006 [17]	MI	n-3 PUFA post-MI	· MI	Payer	3.5 years
			· Stroke		
Liebl et al. 2006 [18]	IGT	Acarbose	· Angina pectoris	Payer	3.3 years
			· Heart failure		
			· MI		
			· Stroke		
Mittendorf et al. 2009 [19]	Type 2 DM	Exenatide	· Angina pectoris	Payer	10 years
			· Blindness		
			· Cataract		
			· Diabetic foot syndrome		
			· End-stage renal disease		
			· Heart failure		
			· MI		
			· Neuropathy		
			· Retinopathy		
			· Stroke		
Neeser et al. 2006 [20]	Atrial fibrillation	Oral vitamin K antagonists	· Stroke	Not specified	10 years
Rasch et al. 2009 [21]	Smoking	Varenicline	· Stroke	Payer	Lifetime
Rosery et al. 2006 [22]	Secondary hyperparathyroidism during haemodialysis	Paricalcitol (i.v.)	· End-stage renal disease	Payer	1 year
Roze et al. 2006 [23]	Insulin-naïve type 2 DM	Acarbose + diet	· Angina pectoris	Payer	35 years
			· Blindness		
			· Cataract		
			· Diabetic foot syndrome		
			· End-stage renal disease		
			· Heart failure		
			· MI		
			· Neuropathy		
			· Stroke		
Schaufler 2009 [24]	Type 2 DM	Type 2 DM prevention	· Blindness	Payer	1 year
			· Diabetic foot syndrome		
			· End-stage renal disease		
			· MI		
			· Retinopathy		
			· Stroke		
Scherbaum et al. 2009 [25]	Type 2 DM with macrovascular disease	Pioglitazone	· Blindness	Payer	35 years
			· Cataract		
			· Diabetic foot syndrome		
			· End-stage renal disease		
			· Heart failure		
			· MI		
			· Neuropathy		
			· Retinopathy		
			· Stroke		
Schwander et al. 2009 [26]	CVD	Eprosartan	· Angina pectoris	Payer	Lifetime
			· MI		
			· Stroke		
Valentine et al. 2008 [27]	Type 2 DM	Insulin detemir ± oral antidiabetic agents	· Angina pectoris	Payer	35 years
			· Blindness		
			· Cataract		
			· Diabetic foot syndrome		
			· End-stage renal disease		
			· Heart failure		
			· MI		
			· Neuropathy		
			· Stroke		
Weber et al. 2007 [28]	Type 2 DM	Self-measurement of blood glucose	· Blindness	Payer	8 years
			· Cataract		
			· Diabetic foot syndrome		
			· End-stage renal disease		
			· Heart failure		
			· MI		
			· Neuropathy		
			· Retinopathy		
			· Stroke		

**ACS** = acute coronary syndromes; **ASA** = acetylsalicylic acid; **CVD** = cardiovascular disease; **DM** = diabetes mellitus; **IGT** = impaired glucose tolerance; **i.v.** = intravenous; **MI** = myocardial infarction; **PCI** = percutaneous coronary intervention; **PUFA** = polyunsaturated fatty acids; **STEMI** = ST-elevation myocardial infarction.

### Information extraction and analysis

In total, 199 costs for clinical events were identified. Details on costs for acute MI and stroke are shown in Table 2 and Table 3, respectively; details on costs for all other clinical events are provided in Additional file 2. The year of costing and the time horizon covered were available for 194 (97%) and 163 (82%) costs, respectively. The cost components covered (e.g., hospitalization, rehabilitation) were specified for only 32 costs (16%); the inflation rate applied was not reported in any of the studies. In most cases (*n* = 140 [70%]), the costs were based on a single literature source. Multiple sources were cited for 32 costs; however, information on data synthesis was not reported. Sources were not stated for 27 costs (14%). Journal articles were the most common source for the derivation of costs (*n* = 153 [77%]), followed by healthcare system reports (*n* = 30 [15%]) and web pages (*n* = 24 [12%]). Of the 35 journal articles that were cited, 19 were cost-of-illness studies (54%).

**Table 2:** Costs for acute myocardial infarction, 2003–2007, Germany

**Study**	**Model type**	**Specification of disease severity**	**First cycle**			**Costs covered**				**Follow-up cycles**		
			**Time horizon**	**Unit cost (€)**	**Number of sources**^**a**^	**Direct costs**^**b**^			**Not specified**	**Time horizon**	**Unit cost (€)**	**Number of sources**^**a**^
						**Hosp**	**Rehab**	**Other**				
**Year of costing: 2003**												
Annemans L. Int J Clin Pract 2006; 60(9): 1129–37 [8]	Markov model	Fatal MI	Not specified	2,880	1				Yes	No follow-up costs considered		
Lamotte M. Pharmacoeconomics 2006; 24(2): 155–69 [16]	Markov model	Fatal MI	Not specified	2,880	NA	Yes				No follow-up costs considered		
Annemans L. Int J Clin Pract 2006; 60(9): 1129–37 [8]	Markov model	Non-fatal MI	Not specified	3,123	1				Yes	Annual follow-up costs	1,907	1
Lamotte M. Pharmacoeconomics 2006; 24(2): 155–69 [16]	Markov model	Non-fatal MI	Not specified	3,123	NA	Yes				Annual follow-up costs	1,907	1
Liebl A. Gesund ökon Qual Manag 2006; 11: 105–11 [18]	Decision tree	MI	Not specified	5,878	1	Yes				Follow-up: rehabilitation	1,261	> 1
										Annual follow-up costs	1,012	> 1
**Year of costing: 2004**												
Lamotte M. Pharmacoeconomics 2006; 24(8): 783–95 [17]	Decision tree	Fatal MI	Acute period	2,880	1	Yes				No follow-up costs considered		
		Non-fatal MI	Acute period	3,123	1	Yes				No follow-up costs considered		
Gandjour A. Health Policy 2007; 83(2–3): 257–67 [14]	Markov model	MI	First 12 months	4,618	> 1				Yes	Post-year 1	2,014	> 1
Brüggenjürgen B. Eur J Health Econ 2007; 8(1): 51–7 [12]	Markov model	MI	First 12 months	11,241	1	Yes	Yes	Yes		Annual follow-up costs	1,006	1
Roze S. Curr Med Res Opin 2006; 22(7): 1415–24 [23]	Semi-Markov model	MI	Year of event	15,011	> 1				Yes	Annual follow-up costs	1,168	1
**Year of costing: 2005**												
Scherbaum WA. Cost Eff Resour Alloc 2009; 7: 9 [25]	Semi-Markov model	Silent MI	Year of event	0	NA				Yes	No follow-up costs considered		
Berg J. Clin Ther 2007; 29(6): 1184–202 [9]	Decision tree and Markov model	MI	Month 1	6,799	> 1				Yes	Months 2–12	5,129	1
Berger K. Curr Med Res Opin 2008; 24(1): 267–74 [11]	Markov model	MI	Initial treatment	7,522	1			Yes		First 6 months after event	2,235	1
										Second 6 months after event	1,484	1
										Subsequent 6- month intervals	759	1
Scherbaum WA. Cost Eff Resour Alloc 2009; 7: 9 [25]	Semi-Markov model	Excluded silent MI	Year of event	8,635	1				Yes	Annual follow-up costs	3,647	1
Weber CJ. Diabetes Sci Technol 2007; 1(5): 676–84 [28]	Markov model	MI	Year of event	16,767	1				Yes	Year after event	1,253	1
**Year of costing: 2006**												
Valentine WJ. Adv Ther 2008; 25(6): 567–84 [27]	Semi-Markov model	MI	Year of event	15,816	> 1				Yes	Annual follow-up costs	1,230	> 1
Schaufler TM. Gesund ökon Qual Manag 2009; 14: 71–5 [24]	Markov model	MI	First 12 months	17,556	1				Yes	Annual follow-up costs	2,323	1
Berg J. Curr Med Res Opin 2008; 24(7): 2089–101 [10]	Decision tree and Markov model	MI	Month 1	6,899	> 1				Yes	Months 2–12	5,204	1
										Annual follow-up costs	2,035	1
**Year of costing: 2007**												
Mittendorf T. Diabetes Obes Metab 2009; 11(11): 1068–79 [19]	Semi-Markov model	MI	Year of event	8,614	1				Yes	Annual follow-up costs	1,292	NA
Schwander B. Value Health 2009; 12(6): 857–71 [26]	Markov model	MI	First 12 months	11,683	> 1				Yes	Annual follow-up costs	2,803	> 1

^a^ Number of data sources from which unit costs were retrieved.

^b^ No indirect unit costs could be retrieved.

**hosp** = hospital*;***MI** = myocardial infarction; **NA** = not available; **rehab** = rehabilitation.

**Table 3:** Costs for stroke, 2003–2007, Germany

**Study**	**Model type**	**Specification of disease severity**	**First cycle**			**Costs covered**				**Follow-up cycles**		
			**Time horizon**	**Unit cost (€)**	**Number of sources**^**a**^	**Direct costs**^**b**^			**Not specified**	**Time horizon**	**Unit cost (€)**	**Number of sources**^**a**^
						**Hosp**	**Rehab**	**Other**				
**Year of costing: 2003**												
Annemans L. Int J Clin Pract 2006; 60(9): 1129–37 [8]	Markov model	Fatal stroke	Not specified	1,897	1				Yes	No follow-up costs considered		
Lamotte M. Pharmacoeconomics 2006; 24(2): 155–69 [16]	Markov model	Fatal stroke	Acute period	1,897	NA	Yes				No follow-up costs considered		
Annemans L. Int J Clin Pract 2006; 60(9): 1129–37 [8]	Markov model	Non-fatal stroke	Not specified	3,390	1				Yes	Annual follow-up costs	676	1
Lamotte M. Pharmacoeconomics 2006; 24(2): 155–69 [16]	Markov model	Non-fatal stroke	Not specified	3,390	NA	Yes				Annual follow-up costs	676	1
Liebl A. Gesund ökon Qual Manag 2006; 11: 105–11 [18]	Decision tree	Stroke	First 3 months	12,068	1				Yes	Months 4–12	1,491	1
										Annual follow-up costs	520	> 1
**Year of costing: 2004**												
Lamotte M. Pharmacoeconomics. 2006; 24(8): 783–95 [17]	Decision tree	Stroke	Acute period	3,390	1	Yes				No follow-up costs considered		
Roze S. Curr Med Res Opin 2006; 22(7): 1415–24 [23]	Semi-Markov model	Fatal stroke	Not specified	9,006	1				Yes	No follow-up costs considered		
Rasch A. Suchtmed 2009; 11(2): 47–55 [21]	Markov model	Stroke	First 12 months	10,149	> 1				Yes	Annual follow-up costs	4,364	> 1
Brüggenjürgen B. Eur J Health Econ 2007; 8(1): 51–7 [12]	Markov model	Stroke	First 12 months	17,734	1	Yes	Yes	Yes		Annual follow-up costs	5,614	1
Roze S. Curr Med Res Opin 2006; 22(7): 1415–24 [23]	Semi-Markov model	Stroke	Year of event	19,399	1				Yes	Annual follow-up costs	6,060	1
Gandjour A. Health Policy 2007; 83(2–3): 257–67 [14]	Markov model	Stroke	First 12 months	24,936	> 1				Yes	Post-year 1	5,465	> 1
**Year of costing: 2005**												
Scherbaum WA. Cost Eff Resour Alloc 2009; 7: 9 [25]	Semi-Markov model	TIA	Year of event	2,354	NA	Yes				Annual follow-up costs	0	NA
Claes C. Med Klin 2008; 103: 778–87 [13]	Markov model	Fatal stroke	Acute period	2,500	NA	Yes				No follow-up costs considered		
Claes C. Med Klin 2008; 103: 778–87 [13]	Markov model	Stroke	Acute period	7,000	NA	Yes				Rehab after acute event	7000	NA
Scherbaum WA. Cost Eff Resour Alloc 2009; 7: 9 [25]	Semi-Markov model	Stoke	Year of event	10,524	> 1				Yes	Annual follow-up costs	6,178	> 1
Weber CJ. Diabetes Sci Technol 2007; 1(5): 676–84 [28]	Markov model	Stroke	Year of event	20,811	1				Yes	Year after event	6,501	1
Berger K. Curr Med Res Opin 2008; 24(1): 267–74 [11]	Markov model	Stroke	Initial treatment	4,692	NA				Yes	First 6 months after event	6,664	NA
		Stroke								Second 6 months after event	5,936	NA
		Stroke								Subsequent 6- month intervals	5,251	
Berg J. Clin Ther 2007; 29(6): 1184–202 [9]	Decision tree and Markov model	Stroke	Month 1	6813	1				Yes	Months 2–12	12,112	1
		Stroke								Annual follow-up costs	5,600	1
**Year of costing: 2006**												
Valentine WJ. Adv Ther 2008; 25(6): 567–84 [27]	Semi-Markov model	Fatal stroke	Not specified	9,488	1				Yes	No follow-up costs considered		
Schaufler TM. Gesund ökon Qual Manag 2009; 14: 71–5 [24]	Markov model	Stroke	First 12 months	18,649	1				Yes	Annual follow-up costs	4,416	1
Valentine WJ. Adv Ther 2008; 25(6): 567–84 [27]	Semi-Markov model	Stroke	Year of event	20,439	1				Yes	Annual follow-up events	6,385	1
Berg J. Curr Med Res Opin 2008; 24(7): 2089–101 [10]	Decision tree and Markov model	Stroke	Month 1	6,912	> 1				Yes	Months 2–12	12,289	1
		Stroke								Annual follow-up costs	5,681	1
**Year of costing: 2007**												
Schwander B. Value Health 2009; 12(6): 857–71 [26]	Markov model	TIA	First 12 months	3,365	> 1				Yes	Annual follow-up costs	0	NA
		Stroke	First 12 months	17,629	> 1				Yes	Annual follow-up costs	7,337	> 1
Mittendorf T. Diabetes Obes Metab 2009; 11(11): 1068–79 [19]	Semi-Markov model	Fatal stroke	Year of event	19,534	1				Yes	No follow-up costs considered		
		Stroke	Year of event	19,534	1				Yes	Annual follow-up costs	5,780	1
**Year of costing: not specified**												
Neeser K. J Kardiol 2006; 13: 131–20 [20]	Markov model	Fatal ischaemic stroke	Not specified	3,573	1				Yes	No follow-up costs considered		
		Severe bleeding	Not specified	9,000	> 1				Yes	After initial hospitalization until end of first year	5,003	1
		Ischaemic stroke	Acute period	4,679	1	Yes				From second year onwards	12,500	1

^a^ Number of data sources from which unit costs were retrieved.

^b^ No indirect unit costs could be retrieved.

**hosp** = hospital; **rehab** = rehabilitation; **NA** = not available; **TIA** = transient ischaemic attack.

### Costs for clinical events

There was considerable variation among the publications in the costs allocated to the clinical events of interest. This variation remained even when findings were filtered to group similar studies together. Table 4 shows the differences in the minimum and maximum costs cited for the first cycle and follow-up cycles in Markov models that had a cycle length of 12 months, and which excluded costs for fatal events, transient ischaemic attack and silent MI. Costs for each clinical event are considered in more detail below.

**Table 4:** Costs for clinical events used in Markov models with a cycle length of 12 months

**Health state**	**First cycle**			**Follow-up cycles**		
	**n**^**a**^	**Minimum (€)**	**Maximum (€)**	**n**^**a**^	**Minimum (€)**	**Maximum (€)**
Acute myocardial infarction	9	4,618	17,556	9	1,006	3,647
Angina pectoris	4	3,342	6,840	4	1,315	6,840
Stroke	10	10,149	24,936	10	676	7,337
Heart failure	4	2,859	6,291	3	800	2,859
Microalbuminuria/macroalbuminuria	0	—	—	0	—	—
End-stage renal disease: renal transplantation	5	45,636	76,135	5	9,129	11,448
Blindness	4	8,685	11,745	2	5,331	10,661
Retinopathy	3	1,862	3,904	1	340	340
Cataract	2	755	1,348	1	0	0
Neuropathy	5	304	4,091	0	—	—
Diabetic foot syndrome: amputation	4	15,405	24,818	2	3,304	3,639

^a^ Number of event costs identified.

Costs for fatal events, silent myocardial infarction and transient ischaemic attack have been excluded.

#### Acute MI

In total, 39 costs for acute MI were reported in 16 studies (Table 2). One study included the cost of a silent MI, which was considered to be zero (0 €) [25]. The cost of an acute MI ranged widely. Even if only those studies that used a Markov model with a cycle length of 12 months were included (*n* = 9), the costs for the first cycle still varied considerably, ranging from 4,618 € to 17,556 € (Table 4) [12,14,19,23-28].

#### Angina pectoris

Five studies reported a total of five costs for angina pectoris (Additional file 2) [18,19,23,26,27]. First cycle costs varied widely, ranging from 3,274 € to 6,840 €. The variation in costs persisted when only those studies that used a Markov model with a cycle length of 12 months were included (Table 4).

#### Stroke

The largest number of costs was reported for stroke: 19 studies reported a total of 55 costs (Table 3). Large variations were observed in the costs attributed to both the first and follow-up cycles for a non-fatal stroke (i.e., excluding fatal events and transient ischaemic attack). Even when only those studies that used a Markov model with a cycle length of 12 months were included, costs still varied considerably, ranging from 10,149 € to 24,936 €, and 676 € to 7,337 €, for the first and follow-up cycles, respectively (Table 4).

#### Heart failure

Six studies reported a total of seven costs for heart failure (Table 4; Additional file 2) [18,19,23,25,27,28]. When only costs from Markov models were included, and non-serious heart failure or hospitalization-only costs were excluded, the costs for the first and follow-up cycles ranged from 2,859 € to 6,291 € and 800 € to 4,372 €, respectively [19,23,27,28]; the cost components for these studies were not specified.

#### Microalbuminuria and macroalbuminuria

No studies reported costs for microalbuminuria or macroalbuminuria.

#### End-stage renal disease

Overall, 31 costs for end-stage renal disease were reported across eight studies (Additional file 2) [15,19,22-25,27,28]. Costs were reported for several different levels of disease severity: renal transplantation, re-transplantation, dialysis, haemodialysis and peritoneal dialysis. End-stage renal disease had the highest reported costs of all of the clinical events evaluated in this review. In most cases, the cost components covered were not specified. The variation could not be accounted for by differences in disease severity, as it persisted when costs were categorized by transplantation (first-cycle costs 45,636–76,135 €) [19,23-25,27] or dialysis (46,296–63,696 €) [19,23-25,27,28]. However, costs for follow-up cycles were consistently lower in transplantation patients than in dialysis patients (9,129–13,176 € vs. 46,296–61,230 €).

#### Blindness

Eleven costs for blindness were reported across six studies (Additional file 2) [19,23-25,27,28]. Costs ranged from 8,685 € to 11,745 € for the first cycle, and from 5,092 € to 11,017 € for subsequent follow-up cycles. The variation in costs persisted when only those studies that used a Markov model with a cycle length of 12 months were included (Table 4).

#### Retinopathy

Only three studies gave costs for retinopathy (Additional file 2) [24,25,28]. Costs for the first cycle were 1,862–3,904 €. Only one study reported costs for follow-up cycles (340 € for hospitalization) (Table 4) [25].

#### Cataract

Costs for cataract varied considerably (Table 4; Additional file 2) [19,23,25,27,28]. However, most studies did not specify the time horizon, thereby limiting the usefulness of cross-study comparisons. In addition, the cost components covered were not usually specified. Generally, studies cited only a single reference for unit cost. Two studies gave costs for hospitalization (755–1,686 €), but neither cited a source [19,28].

#### Neuropathy

Five studies reported costs for neuropathy [19,23,25,27,28] (Additional file 2). With one exception (first cycle 304 €), the costs were fairly consistent (first cycle of other four studies, 3,855–4,091 €), however, the cost components covered were not specified.

#### Diabetic foot syndrome

In total, 21 costs were reported for diabetic foot syndrome, across six studies (Additional file 2) [19,23-25,27,28]. Costs were specified for various disease severities, including gangrene, infected ulcer, uninfected ulcer, healed ulcer and amputation. Costs were almost all based on single references, and generally increased with disease severity (first-cycle costs: healed ulcer, 46–47 € [23,27]; uninfected ulcer, 877–1,210 € [19,23,25,27]; infected ulcer, 1,784–5,217 € [19,23,25,27]; and gangrene, 3,186–13,056 €) [19,23,25,27]. In studies that used a Markov model with a cycle length of 12 months, the cost for the first cycle for amputation ranged from 15,405 € to 24,818 € (Table 4). The reasons for the variability in costs are unclear, as the cost components covered were not specified.

## Discussion

This first systematic review of costs for clinical events used in health economic evaluations in Germany shows substantial cost variability. Exact reasons for these differences are unclear, because of the generally insufficient level of methodological information regarding source data for costing of events in published health economic evaluations. The costs for clinical events published in German health economic analyses are therefore insufficient for establishing future standard lists for such events.

Our findings are consistent with the conclusions of Ray *et al.* that there is considerable internal heterogeneity in both costs and clinical values in countries lacking an established nationwide system of reporting health outcomes [29]. Our observation that the derivation of costs is generally not explicitly defined in German analyses was also consistent with a review of published economic evaluations available in the European Network of Health Economics Evaluation Database [30]. Ideally, event costs should be derived from a robust cost-of-illness study conducted in the same patient population country and setting, and with the same disease severity and treatment algorithm, as the economic evaluation. Typically, however, published cost-of-illness studies do not meet these criteria, and the derived event costs are adjusted for local differences (for example, in treatment pattern, definition of clinical event and disease severity). While this is an acceptable approach, it is essential that the methodology for adjusting the original event costs is clearly defined and supported, and that cost corrections for inflation and currency conversion are performed. Our review shows that most event costs are not based on comprehensive cost-of-illness studies, but on previous cost-effectiveness or cost-utility analyses. While this is by far the simplest approach to obtaining event costs, it raises issues as to whether the previously published cost is appropriate for the new analysis, and was itself derived appropriately.

Accurate and consistent assessment of costs is essential in countries such as Germany that are moving towards early benefit evaluations and cost–benefit analyses of new products relative to current standards of care; hence, there is a clear need to consider how the methodology for estimating costs of clinical events could be improved to ensure that health economic evaluations provide robust, realistic and comparable outputs. What methods can be applied to achieve consensus on costs for clinical events? It is clear that guidance on the derivation of costs should be fully transparent, updated regularly and in the public domain. Three alternatives can be considered.

### Standard cost lists for most relevant medical/non-medical goods or services only

Standard lists of agreed unit costs for medical/non-medical goods or services are provided by multiple countries, including Australia and the Netherlands, and are accompanied by guidance documents on the fundamental process for costing of events [2,5]. This flexible approach enables the evaluation of event costs according to different perspectives and patient subgroups. In countries where healthcare systems are decentralized, cost lists may need to be developed within each major region rather than at a national level; a study in Canada showed the difficulties in consolidating cost lists across two provinces [3].

### Standard cost models for relevant clinical events, in addition to standard cost lists for the most relevant medical/non-medical goods or services

A similar approach is taken by the UK National Health Service (NHS), which publishes detailed annual reports of reference costs [31]. The UK National Institute for Health and Clinical Excellence (NICE) mandates that economic evaluations performed for UK Health Technology Assessment describe how the clinical management of the condition is currently costed in the NHS in terms of these reference costs, and with regard to the relevant Healthcare Resource Group (HRG). This framework for developing costs of clinical events utilizing reference data provides some degree of standardization; however, it is binding only for submissions to NICE, and does not necessarily apply to independent economic evaluations that may ultimately influence decision-making. Moreover, it does not cover resource utilization or costs where the HRG is inappropriate (e.g., too broad or not reflective of resource use related to the treatment in question); in these cases, the NICE guidance document suggests using other sources of evidence, such as micro-costing studies. Such cost data may be taken from current literature, provided that the methods used to identify sources are clearly defined, and that sensitivity analysis are used to assess the implications of using alternative data sources [32]. A recent systematic review of primary research studies funded by the UK HTA programme showed that the majority of studies (72 out of 95 evaluated) obtained source costs from the annual compendium of Unit Costs of Health and Social Care [33]; 52 studies used costs that had been sourced locally (mainly NHS trusts) and 36 of the 95 studies used data from HRGs [34].

### Standard cost lists for relevant clinical events, in addition to standard cost lists for the most relevant medical/non-medical goods or services

A recent feasibility study confirmed the potential for developing standardized diagnosis-specific cost lists for clinical events based on publicly available actual expenditures in the German healthcare system [35]. Costs were derived from the Information System of the Federal Health Monitoring, utilizing the fact that official 2006 cost-of-illness accounts (http://www.gbe-bund.de) provide healthcare expenditures categorized according to the Organisation for Economic Co-operation and Development health accounts (ambulatory care, stationary/semi-stationary healthcare, ambulance services, administration, other providers and private households), as well as according to the three-digit codes of the International Statistical Classification of Diseases and Related Health Problems 10^th^ revision. Although increased data availability from Federal Health Monitoring will likely lead to expansion of these standard lists, this approach may prove too inflexible to take into account the divergent nature of patient populations and perspectives considered in health economic evaluations.

The strengths and limitations of the present study must be considered. Key strengths are that this was a systematic review of all available evidence, considering a clearly defined set of clinical events (related to diabetes mellitus) and studies (cost-utility and cost-effectiveness analyses) conducted from a German perspective. The analysis had a limited focus, in that it included only fully published economic analyses reporting an ICER (i.e., cost-of-illness studies were excluded); however, this was intentional in order to reflect the decision-maker perspective. A number of studies were excluded from the analysis on the basis that the results had only been published in abstract form, rather than as a full text article, indicating that there is limited access to details of country-level health economic evaluations. Other limitations include the fact that our analysis considered only 11 events within a single disease area. Although it seems reasonable to expect that the observed variability in event costs may be generalized to other disease areas, this cannot be concluded with certainty from the current study. Moreover, it was often difficult to judge whether comparisons were fair because of the lack of clarity over exactly how individual studies assessed costs and the nature of their data sources.

## Conclusions

This first systematic review of costs used in health economic evaluations in Germany shows substantial variability in costs for individual clinical events, and a lack of transparency and consistency in the methods used to derive those costs. Ideally, the derivation of costs for clinical events should be fully transparent, based on actual expenditure, updated regularly and in the public domain. While a full standard cost list for clinical events would not be constructive because it is unclear how costs for events vary according to patient group, perspective, and other relevant factors such as knowledge and experience in the therapeutic field of interest, the validity and cross-comparability of health economic evaluations would be considerably improved by national- or regional-level guidance on standardization of the costing methodology of individual clinical events.

## **Additional files**

## Competing interests

Monika Scheuringer and Karl J Krobot are employees of MSD Sharp & Dohme GmbH.

## Authors' contributions

MS was involved in the design and performance of the systematic review and analysis, and in drafting, revision, review and approval of the manuscript. NS was involved in the design and performance of the systematic review and analysis, and in drafting, revision, review and approval of the manuscript. KK was involved in the design of the systematic review and analysis, and in drafting, revision, review and approval of the manuscript. VU was involved in the drafting, revision, review and approval of the manuscript. All authors read and approved the final manuscript.

## Supplementary Material

Additional file 1 **Web Appendix 1.** List of databases and search terms. Click here for file

Additional file 2: **Web Appendix 2.** Costs for clinical events other than acute MI or stroke. Click here for file
