# Supplementary material for: Cost of clinical events in health economic evaluations in Germany: a systematic review
Source: Cost Eff Resour Alloc. 2012 May 31;10:7. doi: 10.1186/1478-7547-10-7 (PMC3495193; doi:10.1186/1478-7547-10-7)
Supplement: Additional file 1 — Web Appendix 1. List of databases and search terms. [file 1478-7547-10-7-S1.pdf]

Web Appendix 1. List of databases and search terms

List of databases searched

Name
Adis Newsletters
BIOSIS Previews
CCMed
Cochrane Library-CDSR
Cochrane Library-Central
DAHTA-Datenbank
Derwent Drug File
Embase
ETHMED
gms
HECLINET
Hogrefe-Verlagsdatenbank und Volltexte
Karger-Verlagsdatenbank
Kluwer-Verlagsdatenbank
Krause & Pachernegg Verlagsdatenbank
MEDIKAT
MEDLINE
NHS-CRD-HTA
SciSearch
SOMED
Springer-Verlagsdatenbank
Springer-Verlagsdatenbank-PrePrint
Thieme-Verlagsdatenbank
Thieme-Verlagsdatenbank-PrePrint

List of search terms

**MODUL Health States**	
#1	*acute myocardial infarction<br><br>("infarction"[MeSH Terms] OR "infarction"[All Fields] OR "infarct"[All Fields]) OR ("myocardial infarction"[MeSH Terms] OR ("myocardial"[All Fields] OR "infarction"[All Fields]) OR "myocardial infarction"[All Fields]) OR "heart attack"[All Fields] OR "ischemic heart disease"[All Fields] OR "myocardial ischemia"[MeSH Terms] OR "myocardial ischemia"[All Fields]

*stroke

("stroke"[MeSH Terms] OR "stroke"[All Fields]) OR "ischemic stroke"[All Fields] OR "hemorrhagic stroke"[All Fields] OR "transient ischemic attack"[All Fields]

*heart insufficiency

"congestive heart failure"[All Fields] OR "heart failure"[All Fields] OR "heart failure"[MeSH Terms] OR "congestive heart failure"[MeSH Terms]

*angina pectoris

"angina pectoris"[MeSH Terms] OR "angina"[All Fields] OR "pectoris"[All Fields] OR "angina pectoris"[All Fields] OR "stenocardia"[MeSH Terms] OR "stenocardias"[All Fields]

*renal insufficiency

"renal disease"[All Fields] OR "renal failure"[All Fields] OR "kidney disease"[All Fields] OR "chronic kidney failure"[All Fields] OR "chronic renal failure"[All Fields] OR "end stage renal disease"[All Fields] OR "nephropathy"[All Fields] OR "diabetic nephropathies"[MeSH Terms] OR "renal transplant"[All Fields] OR "dialysis"[All Fields]

*microalbuminuria/macroalbuminuria

"microalbuminuria"[All Fields] OR ("albuminuria"[MeSH Terms] OR "albuminuria"[All Fields]) OR "macroalbuminuria"[All Fields] OR "macroalbuminuria"[MeSH Terms]

*retinopathy

"retinal diseases"[MeSH Terms] OR "retinal diseases"[All Fields] OR "retinopathy"[All Fields] OR "diabetes retinopathy"[All Fields] OR "diabetic retinopathy"[All Fields] OR "macular retinopathy"[All Fields]

*cataract

("cataract"[MeSH Terms] OR "cataract"[All Fields]) OR "cataract blindness"[All Fields] OR "cataract glaucoma"[All Fields] OR "cataract"[MeSH Terms]

*blindness

"blindness"[MeSH Terms] OR "blindness"[All Fields] OR "severe vision loss"[All Fields]

*amputation owing to diabetic foot syndrome

"foot ulcers"[All Fields] OR "diabetic gangrene"[All Fields] OR "diabetic foot ulcer"[All Fields] OR

	"diabetic neuropathies"[MeSH Terms] OR "diabetic neuropathies"[All Fields] OR "diabetic foot amputations"[All Fields] OR "diabetic foot complication"[All Fields] OR "peripheral artery occlusion"[All Fields] OR "peripheral artery disease"[All Fields] OR "peripheral artery disease"[All Fields] OR ("foot amputation"[MeSH Terms] OR "foot amputation"[All Fields])<br>*neuropathy<br>"diabetic neuropathies"[MeSH Terms] OR "neuropathies"[All Fields] OR "diabetic neuropathies"[All Fields] OR "neuropathy"[All Fields] OR "diabetic neuropathy"[All Fields]
**MODUL study type**	
#2	"cost utility"[TI] OR "cost benefit"[TI] OR "cost effectiveness"[TI] OR "economics, pharmaceutical"[MeSH Terms] OR "$economic$"[TI] OR "cost consequences"[TI]
**MODUL country**	
#3	Search "german"[All Fields] OR ("germany"[MeSH Terms] OR "germany"[All Fields])
**LIMITER**	
#4	Search ("2005/01/01"[PDAT] : "2009/10/30"[PDAT]) AND "humans"[MeSH Terms] AND (English[lang] OR German[lang])
#5	Search Editorial[ptyp] OR Letter[ptyp]
**MERGE HITS**	
#6	Search #1 and #2 and #3
#7	Search #6 and #4
#8	Search #7 not #5
