# Supplementary material for: Cost of clinical events in health economic evaluations in Germany: a systematic review
Source: Cost Eff Resour Alloc. 2012 May 31;10:7. doi: 10.1186/1478-7547-10-7 (PMC3495193; doi:10.1186/1478-7547-10-7)
Supplement: Additional file 2: — Web Appendix 2. Costs for clinical events other than acute MI or stroke. [file 1478-7547-10-7-S2.pdf]

Web Appendix 2

study	specification of disease severity	FIRST CYCLE			FOLLOW-UP CYCLE(S)°			COST COMPOUNDS COVERED					model type
		time horizon	unit cost	number of sources*	time horizon	unit cost	number of sources*	direct cost: hosp.	direct cost: reha.	direct cost: other	indirect cost	not specified	
**angina pectoris**													
year: **2003**													
Liebl A. Gesund ökon Qual manag. 2006;11:105-11.	angina pectoris	not specified	3,274 €	1				☐	☐	☐	☐	☑	decision tree
year: **2004**													
Roze S. Curr Med Res Opin. 2006 Jul;22(7):1415-24.	angina pectoris	year of event	3,342 €	1	annual follow-up costs	3,342 €	1	☐	☐	☐	☐	☑	semi Markov model
year: **2006**													
Valentine W. J. Adv Ther. 2008 Jun;25(6):567-84.	angina pectoris	year of event	3,521 €	>1	annual follow-up costs	3,521 €	>1	☐	☐	☐	☐	☑	semi Markov model
year: **2007**													
Schwander B. Value Health. 2009 Sep;12(6):857-71.	angina pectoris	first 12 months	4,153 €	>1	annual follow-up costs	1,315 €	>1	☐	☐	☐	☐	☑	Markov model
Mittendorf T. Diabetes Obes Metab. 2009 Nov;11(11):1068-79.	angina pectoris	year of event	6,840 €	1	annual follow-up costs	6,840 €	1	☐	☐	☐	☐	☑	semi Markov model

*number of data sources from which unit costs were retrieved
° if blank: no follow up costs were considered in model

hosp: hospital
reha: rehabilitation facility
acute period - other: month 1
na: not available

study	specification of disease severity	FIRST CYCLE			FOLLOW-UP CYCLE(S)°			COST COMPOUNDS COVERED					model type
		time horizon	unit cost	number of sources*	time horizon	unit cost	number of sources*	direct cost hosp.	direct cost reha.	direct cost other	indirect cost	not specified	
**heart failure**													
year: **2003**													
Liebl A. Gesund ökon Qual manag. 2006;11:105-11.	heart failure	not specified	1,741 €	>1				☐	☐	☐	☐	☑	decision tree
year: **2004**													
Roze S. Curr Med Res Opin. 2006 Jul;22(7):1415-24.	heart failure	year of event	6,034 €	1	annual follow-up costs	800 €	1	☐	☐	☐	☐	☑	semi Markov model
year: **2005**													
Scherbaum W. A. Cost Eff Resour Alloc. 2009;7:9.	need for hospitalization	year of event	2,273 €	1	annual follow-up costs	0 €	na	☑	☐	☐	☐	☐	semi Markov model
	non-serious	year of event	35 €	na				☐	☐	☑	☐	☐	
Weber C. J Diabetes Sci Technol. 2007 Sep;1(5):676-84.	heart failure	year of event	5,684 €	1	year after event	4,372 €	1	☐	☐	☐	☐	☑	Markov model
year: **2006**													
Valentine W. J. Adv Ther. 2008 Jun;25(6):567-84.	heart failure	year of event	6,291 €	>1	annual follow-up costs	838 €	1	☐	☐	☐	☐	☑	semi Markov model
year: **2007**													
Mittendorf T. Diabetes Obes Metab. 2009 Nov;11(11):1068-79.	heart failure	year of onset	2,859 €	1	annual follow-up costs	2,859 €	1	☐	☐	☐	☐	☑	semi Markov model

*number of data sources from which unit costs were retrieved
° if blank: no follow up costs were considered in model

hosp: hospital
reha: rehabilitation facility
acute period - other: month 1
na: not available

study	specification of disease severity	FIRST CYCLE: time horizon	FIRST CYCLE: unit cost	FIRST CYCLE: number of sources*	FOLLOW-UP CYCLE(S)°: time horizon	FOLLOW-UP CYCLE(S)°: unit cost	FOLLOW-UP CYCLE(S)°: number of sources*	COST COMPOUNDS COVERED: direct cost hosp.	direct cost reha.	direct cost other	indirect cost	not specified	model type
**endstage renal disease**													
year: **2004**													
Roze S. Curr Med Res Opin. 2006 Jul;22(7):1415-24.	renal transplantation	year of event	68,175 €	1	annual follow-up costs	10,904 €	1	☐	☐	☐	☐	☑	semi Markov model
	haemodialysis	year of event	58,116 €	1	annual follow-up costs	58,116 €	1	☐	☐	☐	☐	☑	
	peritoneal dialysis	year of event	46,296 €	1	annual follow-up costs	46,296 €	1	☐	☐	☐	☐	☑	
year: **2005**													
Rosery H. Clin Drug Invest. 2006;26(11):629-38.	dialysis	not specified	6,376 €	na				☑	☐	☐	☐	☐	decision tree
Scherbaum W. A. Cost Eff Resour Alloc. 2009;7:9.	renal transplantation	year of event	69,504 €	1	annual follow-up costs	11,117 €	1	☐	☐	☐	☐	☑	semi Markov model
	haemodialysis	year of event	59,249 €	1	annual follow-up costs	59,249 €	1	☐	☐	☐	☐	☑	
	peritoneal dialysis	year of event	47,199 €	1	annual follow-up costs	47,199 €	1	☐	☐	☐	☐	☑	
Weber C. J Diabetes Sci Technol. 2007 Sep;1(5):676-84.	dialysis	year of event	60,836 €	1	year after event	60,836 €	1	☐	☐	☐	☐	☑	Markov model
year: **2006**													
Valentine W. J. Adv Ther. 2008 Jun;25(6):567-84.	renal transplantation	year of event	71,828 €	1	annual follow-up costs	11,448 €	1	☐	☐	☐	☐	☑	semi Markov model
	haemodialysis	annual cost	61,230 €	1	annual cost	61,230 €	1	☐	☐	☐	☐	☑	
	peritoneal dialysis	annual cost	48,777 €	1	annual cost	48,777 €	1	☐	☐	☐	☐	☑	
Schaufler T. M. Gesund ökon Qual manag. 2009;14:71-5.	renal transplantation	first 12 months	76,135 €	1	annual follow-up costs	13,176 €	1	☐	☐	☐	☐	☑	Markov model
Schaufler T. M. Gesund ökon Qual manag. 2009;14:71-5.	dialysis	first 12 months	63,696 €	1				☐	☐	☐	☐	☑	Markov model
year: **2007**													
Mittendorf T. Diabetes Obes Metab. 2009 Nov;11(11):1068-79.	haemodialysis	annual cost	54,292 €	1	annual cost	54,292 €	1	☐	☐	☐	☐	☑	semi Markov model
	peritoneal dialysis	annual cost	54,292 €	1	annual cost	54,292 €	1	☐	☐	☐	☐	☑	
	renal transplantation	year of event	45,636 €	1	annual follow-up costs	9,129 €	na	☐	☐	☐	☐	☑	
Jurgensen J.S. Eur J Health Econ. 2009 Mar 19.	re-transplantation	acute period - other	17,561 €	na				☑	☐	☐	☐	☐	Markov model

*number of data sources from which unit costs were retrieved
° if blank: no follow up costs were considered in model

hosp: hospital
reha: rehabilitation facility
acute period - other: month 1
na: not available

study	specification of disease severity	FIRST CYCLE time horizon	FIRST CYCLE unit cost	FIRST CYCLE number of sources*	FOLLOW-UP CYCLE(S)° time horizon	FOLLOW-UP CYCLE(S)° unit cost	FOLLOW-UP CYCLE(S)° number of sources*	COST COMPOUNDS COVERED direct cost hosp.	direct cost reha.	direct cost other	indirect cost	not specified	model type
**blindness**													
year: **2004**													
Roze S. Curr Med Res Opin. 2006 Jul;22(7):1415-24.	blindness	annual cost	10,457 €	1	annual cost	10,457 €	1	☐	☐	☐	☐	☑	semi Markov model
year: **2005**													
Scherbaum W. A. Cost Eff Resour Alloc. 2009;7:9.	severe vision loss	year of event	10,661 €	1	annual follow-up costs	10,661 €	1	☐	☐	☐	☐	☑	semi Markov model
Weber C. J Diabetes Sci Technol. 2007 Sep;1(5):676-84.	blindness	year of event	11,218 €	1	year after event	5,092 €	1	☐	☐	☐	☐	☑	Markov model
year: **2006**													
Valentine W. J. Adv Ther. 2008 Jun;25(6):567-84.	blindness	annual cost	11,017 €	1	annual cost	11,017 €	1	☐	☐	☐	☐	☑	semi Markov model
Schaufler T. M. Gesund ökon Qual manag. 2009;14:71-5.	blindness	first 12 months	11,745 €	1	annual follow-up costs	5,331 €	1	☐	☐	☐	☐	☑	Markov model
year: **2007**													
Mittendorf T. Diabetes Obes Metab. 2009 Nov;11(11):1068-79.	blindness	year of onset	8,685 €	1				☐	☐	☐	☐	☑	semi Markov model

*number of data sources from which unit costs were retrieved
° if blank: no follow up costs were considered in model

hosp: hospital
reha: rehabilitation facility
acute period - other: month 1
na: not available

study	specification of disease severity	FIRST CYCLE time horizon	FIRST CYCLE unit cost	FIRST CYCLE number of sources*	FOLLOW-UP CYCLE(S)° time horizon	FOLLOW-UP CYCLE(S)° unit cost	FOLLOW-UP CYCLE(S)° number of sources*	COST COMPOUNDS COVERED direct cost hosp.	direct cost reha.	direct cost other	indirect cost	not specified	model type
**retinopathy**													
year: **2005**													
Scherbaum W. A. Cost Eff Resour Alloc. 2009;7:9.	retinal photocoagulation	year of event	1,862 €	na	annual follow-up costs	340 €	na	☑	☐	☐	☐	☐	semi Markov model
Weber C. J Diabetes Sci Technol. 2007 Sep;1(5):676-84.	laser coagulation of the retina	year of event	3,729 €	1				☐	☐	☐	☐	☑	Markov model
year: **2006**													
Schaufler T. M. Gesund ökon Qual manag. 2009;14:71-5.	retinopathy	first 12 months	3,904 €	1				☐	☐	☐	☐	☑	Markov model

*number of data sources from which unit costs were retrieved
° if blank: no follow up costs were considered in model

hosp: hospital
reha: rehabilitation facility
acute period - other: month 1
na: not available

study	specification of disease severity	FIRST CYCLE			FOLLOW-UP CYCLE(S)°			COST COMPOUNDS COVERED					model type
		time horizon	unit cost	number of sources*	time horizon	unit cost	number of sources*	direct cost hosp.	direct cost reha.	direct cost other	indirect cost	not specified	
**cataract**													
year: **2004**													
Roze S. Curr Med Res Opin. 2006 Jul;22(7):1415-24.	laser treatment	not specified	3,477 €	1				☐	☐	☐	☐	☑	semi Markov model
	cataract surgery	not specified	1,322 €	1				☐	☐	☐	☐	☑	
year: **2005**													
Scherbaum W. A. Cost Eff Resour Alloc. 2009;7:9.	cataract surgery	year of event	1,348 €	1				☐	☐	☐	☐	☑	semi Markov model
Weber C. J Diabetes Sci Technol. 2007 Sep;1(5):676-84.	cataract surgery	year of event	755 €	na				☑	☐	☐	☐	☐	Markov model
year: **2006**													
Valentine W. J. Adv Ther. 2008 Jun;25(6):567-84.	laser treatment	not specified	3,663 €	1				☐	☐	☐	☐	☑	semi Markov model
	cataract	not specified	1,385 €	1				☐	☐	☐	☐	☑	
year: **2007**													
Mittendorf T. Diabetes Obes Metab. 2009 Nov;11(11):1068-79.	laser treatment	not specified	2,079 €	1				☐	☐	☐	☐	☑	semi Markov model
	cataract surgery	not specified	1,686 €	na				☑	☐	☐	☐	☐	

*number of data sources from which unit costs were retrieved
° if blank: no follow up costs were considered in model

hosp: hospital
reha: rehabilitation facility
acute period - other: month 1
na: not available

study	specification of disease severity	FIRST CYCLE			FOLLOW-UP CYCLE(S)°			COST COMPOUNDS COVERED					model type
		time horizon	unit cost	number of sources*	time horizon	unit cost	number of sources*	direct cost hosp.	direct cost reha.	direct cost other	indirect cost	not specified	
**neuropathy**													
year: **2004**													
Roze S. Curr Med Res Opin. 2006 Jul;22(7):1415-24.	neuropathy	year of event	3,855 €	na				☑	☐	☐	☐	☐	semi Markov model
year: **2005**													
Scherbaum W. A. Cost Eff Resour Alloc. 2009;7:9.	neuropathy	year of event	3,930 €	1				☐	☐	☐	☐	☑	semi Markov model
Weber C. J Diabetes Sci Technol. 2007 Sep;1(5):676-84.	polyneuropathy	year of event	304 €	1	year after event	304 €	1	☐	☐	☐	☐	☑	Markov model
year: **2006**													
Valentine W. J. Adv Ther. 2008 Jun;25(6):567-84.	neuropathy	first 12 months	4,091 €	1				☐	☐	☐	☐	☑	semi Markov model
year: **2007**													
Mittendorf T. Diabetes Obes Metab. 2009 Nov;11(11):1068-79.	neuropathy	year of onset	4,067 €	1				☐	☐	☐	☐	☑	semi Markov model

*number of data sources from which unit costs were retrieved
° if blank: no follow up costs were considered in model

hosp: hospital
reha: rehabilitation facility
acute period - other: month 1
na: not available

study	specification of disease severity	FIRST CYCLE time horizon	FIRST CYCLE unit cost	FIRST CYCLE number of sources*	FOLLOW-UP CYCLE(S)° time horizon	FOLLOW-UP CYCLE(S)° unit cost	FOLLOW-UP CYCLE(S)° number of sources*	COST COMPOUNDS COVERED direct cost hosp.	direct cost reha.	direct cost other	indirect cost	not specified	model type
**diabetic foot syndrome**													
year: **2004**													
Roze S. Curr Med Res Opin. 2006 Jul;22(7):1415-24.	gangrene	not specified	3,186 €	1				☐	☐	☐	☐	☑	semi Markov model
	infected ulcer	not specified	1,784 €	1				☐	☐	☐	☐	☑	
	uninfected ulcer	not specified	877 €	1				☐	☐	☐	☐	☑	
	healed ulcer	annual cost	46 €	1	annual cost	46 €	1	☐	☐	☐	☐	☑	
year: **2005**													
Scherbaum W. A. Cost Eff Resour Alloc. 2009;7:9.	bypass surgery; revascularization of leg	year of event	6,268 €	na				☑	☐	☐	☐	☐	semi Markov model
	gangrene	year of event	3,248 €	1				☐	☐	☐	☐	☑	
	Infected ulcer	year of event	1,818 €	1				☐	☐	☐	☐	☑	
	uninfected ulcer	year of event	894 €	1				☐	☐	☐	☐	☑	
Weber C. J Diabetes Sci Technol. 2007 Sep;1(5):676-84.	feet ulceration	year of event	990 €	1	year after event	990 €	1	☐	☐	☐	☐	☑	Markov model
year: **2006**													
Valentine W. J. Adv Ther. 2008 Jun;25(6):567-84.	gangrene	not specified	3,356 €	1				☐	☐	☐	☐	☑	semi Markov model
	infected ulcer	not specified	1,879 €	1				☐	☐	☐	☐	☑	
	uninfected ulcer	not specified	924 €	1				☐	☐	☐	☐	☑	
	healed ulcer	annual cost	47 €	1	annual cost	47 €	1	☐	☐	☐	☐	☑	
Schaufler T. M. Gesund ökon Qual manag. 2009;14:71-5.	feet ulceration	first 12 months	1,036 €	1				☐	☐	☐	☐	☑	Markov model
year: **2007**													
Mittendorf T. Diabetes Obes Metab. 2009 Nov;11(11):1068-79.	gangrene	not specified	13,056 €	1				☐	☐	☐	☐	☑	semi Markov model
	infected ulcer	not specified	5,217 €	1				☐	☐	☐	☐	☑	
	uninfected ulcer	not specified	1,210 €	1				☐	☐	☐	☐	☑	
	non standard ulcer treatment	not specified	544 €	1				☐	☐	☐	☐	☑	

*number of data sources from which unit costs were retrieved
° if blank: no follow up costs were considered in model

hosp: hospital
reha: rehabilitation facility
acute period - other: month 1
na: not available

study	specification of disease severity	FIRST CYCLE time horizon	FIRST CYCLE unit cost	FIRST CYCLE number of sources*	FOLLOW-UP CYCLE(S)° time horizon	FOLLOW-UP CYCLE(S)° unit cost	FOLLOW-UP CYCLE(S)° number of sources*	COST COMPOUNDS COVERED direct cost hosp.	direct cost reha.	direct cost other	indirect cost	not specified	model type
**diabetic foot syndrom: amputation**													
year: **2004**													
Roze S. Curr Med Res Opin. 2006 Jul;22(7):1415-24.	amputation	not specified	22,096 €	1				☐	☐	☐	☐	☑	semi Markov model
year: **2005**													
Scherbaum W. A. Cost Eff Resour Alloc. 2009;7:9.	leg amputation (major, above ankle)	year of event	15,405 €	>1	annual follow-up costs	3,304 €	>1	☐	☐	☐	☐	☑	semi Markov model
Weber C. J Diabetes Sci Technol. 2007 Sep;1(5):676-84.	amputation	year of event	23,704 €	1	year after event	3,476 €	1	☐	☐	☐	☐	☑	Markov model
year: **2006**													
Valentine W. J. Adv Ther. 2008 Jun;25(6):567-84.	amputation	not specified	23,280 €	1				☐	☐	☐	☐	☑	semi Markov model
Schaufler T. M. Gesund ökon Qual manag. 2009;14:71-5.	amputation	first 12 months	24,818 €	1	annual follow-up costs	3,639 €	1	☐	☐	☐	☐	☑	Markov model
year: **2007**													
Mittendorf T. Diabetes Obes Metab. 2009 Nov;11(11):1068-79.	amputation	year of event	21,417 €	1				☐	☐	☐	☐	☑	semi Markov model

*number of data sources from which unit costs were retrieved
° if blank: no follow up costs were considered in model

hosp: hospital
reha: rehabilitation facility
acute period - other: month 1
na: not available
